# An Intervention Study: Does a Cognitive Reappraisal Technique Reduce the Perceived Stress in Fourth-Year Dental Students in New Zealand?

**DOI:** 10.1155/2019/5864591

**Published:** 2019-04-01

**Authors:** Alana Smith, Imogen Scott, Jithendra Ratnayake, Kate Newsham-West, Peter Cathro

**Affiliations:** Faculty of Dentistry, The University of Otago, 310 Great King Street, Dunedin 9016, New Zealand

## Abstract

**Background:**

The fourth-year of the Bachelor of Dental Surgery (BDS) degree is considered the most stressful in the curriculum. Cognitive reappraisal is a self-applied method of stress management where an individual recognises his/her physiological responses to stress as a positive phenomenon helping him/her rise to the challenge, rather than a negative one in response to a threat situation.

**Aim:**

To investigate whether teaching fourth-year dental students to apply cognitive reappraisal reduces their perceived levels of stress.

**Methods:**

A survey was emailed to all fourth-year dental students, inviting them to respond to a 20-item questionnaire adapted from the Dental Environmental Stress (DES) Survey. Respondents were randomly assigned to reappraisal intervention/experimental (EXP) and control intervention (CON) groups, and each group was asked to watch an educational video. The EXP group video educated respondents on how to apply cognitive reappraisal in stressful situations, and the CON group video described generic methods of stress management. A follow-up survey was conducted after 3 weeks.

**Results:**

The respondent rate was 47.6%. Change scores were calculated by subtracting the follow-up DES scores from baseline DES scores. The average change score for the experimental group was +3.1, indicating a decrease in average perceived stress levels. Conversely, the average change score for the CON group was −1.06, indicating an increase in average perceived stress levels. However, this difference did not reach a statistical significance.

**Conclusion:**

EXP group has shown to have positive effects on stress management, and its effects on BDS students demonstrate promise.

## 1. Introduction

Stress can be defined as the strain that accompanies a demand (physical or mental), which is perceived as either challenging (positive) or threatening (negative) and can enable adaptation or be debilitating [1]. Stress is a part of most people's lives at some point and can be the main motivation behind many of our grandest achievements [2]. However, it is a two-edged sword, if stress is viewed as a challenge it can motivate someone to reach their peak performance, whereas, if stress is viewed as a threat, it can cripple the individual into ineffectiveness [2]. Eliminating all stressful problems in a dental education programme is difficult, and students have to reach high levels of knowledge and skill, as well as developing good attitudes towards patient care within a short period time to become a successful dental professional. Many studies have highlighted that tertiary training at dental school can be a source of considerable stress and dentistry as a profession is stressful [1, 3]. Studies unanimously report that the stress experienced whilst training at dental school has negative effects on both the mental and physical health of the students such as anxiety, depression, burnout, fatigue, sleeplessness, gastrointestinal symptoms, and irritability [4–6].

It has been reported that the clinical years are considered more stressful than the preclinical years, owing to the additional patient and treatment-related stressors [1, 5, 7]. The Faculty of Dentistry at the University of Otago is the only Faculty and School of Dentistry in New Zealand, and the duration of the Bachelor of Dental Surgery (BDS) is five years. Although the fourth-year of study is not the first year of clinical exposure, it is considered the most stressful year because of the high demand in both complex clinical and academic facets of learning. Generalised stressors of the clinical years were noted to be completing clinical requirements, patient cancellations, atmosphere created by supervisors, heavily loaded days, examinations, grades, and lack of time for relaxation [8]. Students commonly subscribe to a stress-relief mechanism by taking time out for relaxation and recuperation. However, avoiding stress is not plausible for demanding years of study, and it is not an effective life-long stress management strategy. Dental students often do not have time for traditional stress relief through recreation, social interaction, and exercise. Therefore, students may fall into a habit of pursuing unhealthy coping mechanisms such as smoking, alcohol, drug use, and over spending [3, 9]. Cognitive symptoms include feeling frustrated, becoming easily confused, unable to concentrate, having memory problems, thinking negatively, and gastrointestinal problems. Cognitive reappraisal involves recognizing the negative pattern your thoughts have fallen into and changing that pattern to one that is more effective. This is a useful strategy because it can enable individuals to downregulate negative feelings and switch to more positive ones, helping to maximise performance [10]. Effectively, interpretations of bodily signals (e.g., increased heart rate) affect how the body and mind respond to acute stress. If the individual believes that this change is a sign of their body preparing them for action, they will feel a “challenge” response to the situation. Conversely, if they had perceived the increased heart rate as a sign of anxiety and nervousness, they will feel a “threat” response. This suggests that appraisal of stress is critical in determining the impact of the stressor and the consequent reaction and outcome when individuals are faced with a challenge. Challenge is associated with positive physiological outcomes such as increased cardiac efficiency and vasodilation, whereas threat is associated with negative outcomes, reduced cardiac efficiency, and vasoconstriction [10, 11].

In an environment in which stress is inevitable such as the fourth-year of the BDS course, cognitive reappraisal could prove to be invaluable for management of stress in dental students and for their future careers. Several studies have demonstrated that the dental stress questionnaire (DES) is an effective tool to examine the level and sources of stress associated with dental education [6, 7].

The main aim of this current study was to assess whether applying cognitive reappraisal could affect the perceived levels of stress in clinical situations for fourth-year dental students and whether teaching them to apply cognitive reappraisal would reduce their perceived levels of stress using the dental environmental stress questionnaire.

## 2. Method

Ethical approval was granted from Human Ethics Committee of Otago University (16/090). The study population comprised undergraduate fourth-year dental students from the 2016 class (*n* = 84) enrolled in the BDS programme at the University of Otago. The students had to complete an online questionnaire, watch a presentation regarding stress management, and complete a second questionnaire. The students were randomly assigned into two intervention groups: cognitive reappraisal intervention/experimental (EXP) and control intervention (CON) groups.

The level of stress perceived by fourth-year dental students when exposed to different dentally related clinical and academic stressors was assessed using a modified dental environmental stressor (DES) questionnaire introduced by Gambee (1980). The DES questionnaire is a 38-item questionnaire which is used to assess sources of stresses associated with undergraduate course work and training in dental students [12–15]. From the 38 questions, 20 questions were selected from the DES questionnaire being applicable to the New Zealand dental education background (Table 1). The questionnaire which consisted of 20 questions was piloted among 10 current 5^th^ year dental students. The modified DES questionnaire was distributed to the fourth-year students electronically via student email and on the University Intranet (blackboard), and the participants had to fill in a consent form. The questionnaire collected the respondent's student ID numbers for subsequent comparison reasons only. Respondents were asked to indicate the level of stress they perceived in response to the situations outlined using a Likert scale (1, not stressful; 2, somewhat stressful; 3, moderately stressful; 4, highly stressful).

The first online survey collected demographic information (age, gender, and whether English is the first spoken language or not) and the responses to the DES questionnaire. Two public reminders were sent to the fourth-year BDS class encouraging members to complete the questionnaires, and Internet links to the questionnaire were available for a three-week period. Responding students were randomly allocated into cognitive reappraisal intervention/experimental (EXP) and control intervention (CON) groups. The baseline number of respondents in both CON and EXP groups was 20.

Subsequently, the experimental and control groups' respondents were sent their respective videos via private student email, which included a note encouraging fourth-year students to incorporate these methods into their stress management skillset. The experimental group respondents received a link to a 4.5-minute video, which was created using stop-motion animation and outlined the principles of cognitive reappraisal and how its methods can be directly incorporated into the lives of fourth-year dental students. The video consists of several examples of stressful situations fourth-year dental students' experiences such as removing deep caries in patients using burs and exposing the pulp (https://www.youtube.com/watch?v=aekYVSVqxE0&feature=youtu.be).

The control intervention group respondents were sent a link to a 4.5-minute informative video presentation via private student email which was made by BBC Brainsmart and outlined how to manage stress through methods such as taking deep breaths, getting exercise, planning stressful periods of time, socializing, and taking time out to relax. Both groups had three weeks of video availability and time to implement the techniques outlined therein. Access to the video was granted only by using the link sent to students via email, and respondents were firmly instructed not to share the video with any of their classmates. Eight weeks after the baseline survey, the respondents were asked to complete a follow-up DES questionnaire. The questionnaire took the same format as the initial questionnaire in assessment of perceived levels of stress, with the addition of one question, asking, “How many times did you watch the intervention video?” The link to the follow-up questionnaire was available for a two-week period and an email reminder was sent to the nonrespondents.

Participation in this research was on voluntary basis, and no incentives were used for the respondents. Using student ID numbers as identifiers safeguarded confidentiality, and any email sent to the respondents were blind carbon copied, ensuring that the email addresses of the other participants remained private.

## 3. Statistical Analysis

Analysis of all data was carried out using Microsoft Excel software. EXP and CON groups were assessed identically. Follow-up DES stress perception scores were matched to their respective baseline scores for each participant for each scenario. The sum of all baseline and follow-up responses was calculated for each respondent. Change scores were calculated by subtracting the follow-up sum (*∑*) score from the baseline sum (*∑*) score for each scenario. Positive change scores indicate a reduction in the perceived stress levels, and negative change scores indicate an increase in the perceived stress levels. The change scores in CON and EXP groups were compared to assess which DES scenarios had experienced the greatest change in perceived stress score in EXP and CON groups. Student's *t*-test was carried out to compare the change scores on the EXP and CON groups. A *P* value of ≤0.05 was considered statistically significant.

## 4. Results

There were 40 respondents to the initial DES questionnaire with a response rate of 47.6% of the class. The age range for this group was 21–28 years of age and comprised of eleven males (27.5%) and twenty-nine females (72.5%). The baseline responses to the DES questionnaire were not considered significantly different between the EXP and CON groups as the participants were allocated randomly to their respective groups. Follow-up response rate was considered reasonable at 90% (18/20) in CON group and 95% (19/20) in EXP group.  Table 2 outlines the sum of respondents DES scores (∑DES) at baseline and follow-up and the associated change score (Δ in ∑DES) for each respondent. The average baseline ∑DES score for the EXP group was 59.53 and for the CON group 52.39. The average follow-up ∑DES score for the EXP group was 56.42 and for the CON group 53.44. The average change score for the EXP group was +3.11, indicating a decrease in perceived stress levels, whilst the average change score for the CON group was −1.06 indicating an increase in perceived stress score. Results of the present study demonstrate that the EXP group showed a small-to-medium decrease in perceived level of stress in comparison to the CON group, which did not reach statistical significance.

## 5. Discussion

Results of the present study showed that applying cognitive therapy has promising effects on reducing the level of stress perceived by fourth-year dental students. However, the results were not statistically significant (*P*=0.076). The most polarising differences for the average scores for DES scenarios resulted in scenario 4 “completing for graduating requirements,” resulting in +10 for experimental and −3 for control group respectively. In addition, scenario 7 “responsibilities of comprehensive patient care” (+11 for EXP, −1 for CON) and scenario 8 “patients not available for prescribed times of treatment or examination, i.e., cancellations” (+10 for EXP, −2 for CON) resulted in substantial differences (Table 1). The experimental group showed more positive change than the CON group in these scenarios. Interestingly, these three scenarios are connected. As students are more able to undertake comprehensive treatment plans for patients and accept responsibility for their completion, this enables students to carry out the necessary procedures to meet graduation requirements. When cancellations occur, often out of the student's control, this puts both the completion of treatment and of graduation requirements at a risk of failure.

The DES stressor scenario that scored highest in both the CON group and EXP group at baseline was DES 6 “fear of being unable to keep up with workload” and at follow-up was DES 3 “examinations and grading.” This change is representative of the time in the academic year. When the first questionnaire was collected the focus was on completing clinical trials, whereas the focus shifted to examinations when the follow-up questionnaires were collected. In a study conducted in India which investigated perceived sources of stress on dental students, the top stressor for all years was “fear of failing” followed by “fear of unemployment after graduating” and then “financial sources.” Stressors such as “lack of time for relaxation” was a less stressful item for Indian Dental students [16]. This may be largely due to the fact that the education curriculum in India is different from that of New Zealand. New Zealand dental students have a high rate of employment and have access to student loans and student allowances which reduces the burden of financial sources. However, in a similar study in Malaysia, fear of failing the course at the end of year exams; concerns regarding completion of clinical work and examination results and grades were found as top stressors among dental students which is similar to the findings in this study [14]. In an Australian study which has a similar education curriculum to New Zealand, one of the main stressors students faced was the “transition to clinical learning” [1, 12]. Another study conducted in Australia showed that students ranked “examinations and grades” as the single most stress-inducing concerns and the stress intensity peaked in the fourth-year of training [13].

The scenarios experiencing the most positive effect from the cognitive reappraisal intervention (experimental group) were “responsibilities of comprehensive patient care,” “competing for graduation requirements,” and “fear of being unable to keep up with workload.” However, the least positive scenarios were “examinations and grading,” “attitudes of faculty towards professional students,” and “rules and regulations of the school.” This would indicate that the cognitive reappraisal method has the greatest impact on clinical rather than academic or bureaucratic stressful scenarios. By comparison, there was no clear trend in the CON group, for whom the scenarios experiencing the greatest positive change were “lack of time for relaxation,” “lack of time to do assigned work,” “fear of being unable to keep up with workload,” and “difficulty in learning clinical procedures” and the least positive effect was on “competition with classmates.” The change scores for the CON group were overall a lot lower than for the EXP group, indicating that the cognitive reappraisal method changed the perceived stress levels by a greater magnitude and in a positive direction.

The effects of cognitive reappraisal on improving perceived levels of stress has not been investigated thoroughly in the past. Much of the literature uses physiological markers such as cardiovascular stress, salivary alpha amylase, and performance outcomes to measure stress [10]. The majority of the studies reporting on the application of cognitive reappraisal in stressful situations demonstrated an individual can improve these physiological responses to stress, e.g., increased cardiac efficiency, lower vascular resistance, increases in sympathetic nervous system activation, and improved performance [10, 11]. The assumption is that these beneficial effects would be followed by a reduction in perceived stress levels. However, these effects did not translate significantly into reduced perception of stress levels in the DES scores of the EXP group as compared to the CON group.

It is difficult to know if demographic factors such as gender, ethnicity, age, first spoken language, international or domestic status, and having completed a prior degree influenced the outcome of the study. The sample size was not large enough to ensure it was representative of the class's demographic characteristics. Response rate was limited in part due to the small size of the fourth-year BDS class (84 students). Ceilings in response such as students who were either stressed or laid-back to respond were expected. Had these students responded to the survey, there was a high chance that they would have been potential outliers in the data. This is demonstrated by the respondents in the CON group lost to follow-up having the highest DES scores at baseline (indicating they may have been too stressed to respond during the second time). Conversely, the respondent in the EXP group lost to follow-up had the lowest DES score at baseline (indicating they may have been too laid back to respond a second time). Another limitation to baseline and follow-up response rates includes respondent burden. Fourth-year dental students have a consistently heavy workload; therefore, having to fill out two online surveys and watch a video on different occasions can be relatively time consuming and easy to forget. An alternative solution would be to hand out hard copies of the surveys before the start of a fourth-year lecture. Although this may compromise the confidentiality and anonymity of responses, it designates a specific time for respondents to complete the survey without imposing on their personal schedule.

As the baseline and follow-up surveys were conducted eight weeks apart, the fourth-year dental students had time to practice complex dental procedures and participate in ongoing learning. As students gain more experience in their clinics, a dental scenario that may have perceived as stressful in the past may no longer be considered as stressful. This may have attributed to reductions in perceived levels of stress, rather than the effects of cognitive reappraisal improving their management of stress. The type of clinical experiences each student gains throughout the academic year varies and not all students gain the same level of experience. In addition, there might not have adequate time or complex clinical procedures to implement cognitive reappraisal techniques to have a sufficient effect on their perceived levels of stress. To counteract these problems in future research, adequate time should be given between the baseline and follow-up surveys to allow for the application of cognitive reappraisal in a dental environment.

The potential positive effects of cognitive reappraisal may have been limited by the video in which education of the stress management technique was delivered. The videos for both the groups were administered via private email for respondents to view in their own time. This meant distractions could not be controlled for, and the full length of the video may not have been viewed. Similarly, the number of times the videos were viewed was not controlled for. In addition, the participants were not limited to other methods of stress management such as watching psychoeducation videos. Psychoeducation is a cognitive behavioural therapy which provides education and information to those who face psychological stresses on a daily basis [17]. Participants of both groups could have explored and viewed further psychoeducational videos which would have reduced their perceived stress. Although these factors were few limitations of this study, it is difficult to conclude that these factors had an effect on the final result. In a future study, it would be interesting to see whether combining cognitive reappraisal therapy with additional stress management strategies would have a significant effect.

The current literature consistently reports on the high levels of stress experienced by dental students worldwide and expresses a need for programmes or courses for prevention and intervention of stress [18]. However, there are very few studies that measure the efficacy of any of the proposed methods in achieving these aims. This study to the best of our knowledge is the first randomized controlled trial in which cognitive reappraisal has been tested for stress management in dental students. This research paves the way for further investigation into successful implementation of cognitive reappraisal in the dental environment. Other areas of interest include the longevity of a single exposure to a cognitive reappraisal intervention and if ongoing exposure to the intervention is required for positive effects.

## 6. Conclusion

Cognitive reappraisal has been shown to have positive effects on stress management, and its effects on BDS students demonstrate promise. It is a technique that can be applied by fourth-year dental students in the dental environment that does not require removal of stressors to reduce perceived levels of stress. The result of the cognitive reappraisal intervention group was promising; however, there was no statistically significant difference compared to the control group (*P*=0.076). Therefore, further research and adjustment of the intervention could produce more statistically significant improvements and predictable outcomes. The prospective cognitive reappraisal intervention could therefore be justified for implemented in the BDS curriculum to improve the performance and well-being of dental students at the Otago Dental School, a skill that will transcend into their professional careers and beyond.

## Figures and Tables

**Table 1:** DES scores for each scenario in the DES questionnaire.

		Control group			Experimental group			
	DES scenario	Baseline score	Follow-up score	Difference in scenario	Baseline score	Follow-up score	Difference in scenario	Difference in change score
1	Amount of assigned work	55	55	0	65	57	8	8
2	Competition with classmates	33	38	−5	46	45	1	6
3	Examinations and grading	54	58	−4	62	67	−5	−1
4	Competing for graduation requirements	47	50	−3	57	47	10	13
5	Lack of time to do assigned work	57	52	5	57	55	2	−3
6	Fear of being unable to keep up with workload	59	54	5	67	57	10	5
7	Responsibilities of comprehensive patient care	53	54	−1	66	55	11	12
8	Patients not available for prescribed times of treatment or examination	47	49	−2	63	53	10	12
9	Difficulty in learning clinical procedures	46	41	5	58	58	0	−5
10	Difficulty in learning precision skills required in clinical and laboratory work	47	48	−1	60	57	3	4
11	Working on patients with poor OH	37	37	0	48	41	7	7
12	Learning environment created by the faculty	49	48	1	61	61	0	−1
13	Receiving criticism about work	41	45	−4	45	44	1	5
14	Rules and regulations of the school	33	39	−6	38	42	−4	2
15	Attitudes of faculty towards professional students	40	43	−3	48	49	−1	2
16	Inconsistency of feedback on your work among different instructors	57	55	2	65	64	1	−1
17	Lack of time for relaxation	48	43	5	61	53	8	3
18	Lack of confidence to be a dental student	44	44	0	57	55	2	2
19	Insecurity concerning your professional future	52	48	4	58	49	9	5
20	Lack of confidence in career decision	44	40	4	49	41	8	4

**Table 2:** Summary of the DES scores: CON group and EXP group.

	∑DES base	∑DES follow-up	Δ in ∑DES
*CON respondents*			
1	57	61	−4
2	49	50	−1
3	71	65	6
4	46	41	5
5	32	34	−2
6	41	41	0
7	57	52	5
8	43	46	−3
9	63	61	2
10	44	49	−5
11	47	59	−12
12	54	64	−10
13	63	61	2
14	48	50	−2
15	71	64	7
16	65	63	2
17	31	38	−7
18	61	63	−2
Average score	52.39	53.44	−1.06

*EXP respondents*			
1	70	50	20
2	57	65	−8
3	56	39	17
4	54	53	1
5	68	55	13
6	59	63	−4
7	58	47	11
8	52	50	2
9	52	42	10
10	64	71	−7
11	64	58	6
12	67	59	8
13	51	52	−1
14	65	63	2
15	58	65	−7
16	50	50	0
17	58	61	−3
18	61	62	−1
19	67	67	0
Average score	59.53	56.42	3.11

SD	9.88	9.47	—
Difference in average change score			4.16
*T* test (*P* value)			0.076
Difference in average change scores			4.16
Standard deviation base			9.88
Standardised effect size = change in average / base·SD			0.42

## Data Availability

The frequency and percentage data used to support the findings of this study are included within the article.
